# Effects of CNTs/PVA on Concrete Performance: Strength, Drying Shrinkage, and Microstructure

**DOI:** 10.3390/ma18112535

**Published:** 2025-05-28

**Authors:** Shengliang Lu, Ting Zuo, Zhongkun Wang, Shuang Yan

**Affiliations:** 1 School of Civil Engineering & Architecture, Wenzhou Polytechnic, Wenzhou 325035, China; 2009230009@wzpt.edu.cn; 2 School of Construction Engineering, Yunnan Land and Resources Vocational College, Kunming 652501, China; zteducn@163.com; 3 College of Civil Engineering and Architecture, Wenzhou University, Wenzhou 325035, China; wangzhongkun@wzu.edu.cn; 4 Department of Civil Engineering and Smart Cities, Shantou University, Shantou 515063, China

**Keywords:** carbon nanotubes (CNTs), polyvinyl alcohol (PVA), mechanical properties, drying shrinkage, mechanism

## Abstract

A uniformly dispersed carbon nanotubes (CNTs)/polyvinyl alcohol (PVA) nano-colloidal emulsion was synthesized by leveraging colloidal stability and interfacial chemical interactions. This study systematically investigated the influence of the CNTs/PVA nano-colloidal emulsion on the mechanical properties, drying shrinkage, capillary water absorption, and microstructure of cement-based materials, while elucidating the underlying reinforcement mechanisms. The experimental results demonstrated that different CNTs/PVA ratios enhanced the concrete properties: For instance, 0.3% CNTs and 1.0% PVA improved the 28-day compressive and flexural strengths by 15% and 10%, respectively, while 0.5% CNTs and 1.0% PVA reduced the drying shrinkage by 76%, 34%, 22%, and 21% at 7, 28, 180, and 360 days. Additionally, the 0.5% CNTs/1.0% PVA mixture achieved a 25.7% lower absorption rate (25.25 vs. 34.00 g·m^−2^, *p* < 0.001) than plain concrete. A microstructural analysis revealed that the CNTs/PVA composite formed an interpenetrating network within the cement matrix, which correlated with the observed mechanical improvements and shrinkage reduction. These findings indicate that even minimal additions of CNTs/PVA could effectively enhance the tensile and flexural capacity of concrete while mitigating its susceptibility to drying shrinkage.

## 1. Introduction

Cement concrete is the most widely used construction material in the world, and its performance directly impacts the safety and durability of buildings. There are numerous pores and microcracks inside cement concrete, and the moisture within varies with the environmental temperature and humidity, leading to drying shrinkage deformation or even cracking [1]. The presence of cracks significantly compromises the safety, durability, and service life of concrete structures and buildings. The drying shrinkage of concrete is primarily related to the migration amount and rate of physically adsorbed water in calcium silicate hydrate (C-S-H) and capillary pore-adsorbed water [2]. Generally, the more pores and cracks in the concrete, the greater the migration amount and rate of adsorbed water. Fibers can inhibit crack formation, thereby reducing concrete shrinkage [3,4,5].

As a nanoscale material, carbon nanotubes (CNTs) far surpass any conventional fiber in terms of the elastic modulus, strength, and toughness. CNTs exhibit excellent pore-filling properties, significantly reducing the concrete porosity, improving the pore structure, and decreasing the water absorption [6,7,8]. More importantly, due to their extremely high aspect ratio, even a minimal amount of CNTs can substantially enhance various properties of concrete [9,10]. However, CNTs face challenges, such as a large specific surface area, high surface energy, and strong entanglement tendencies, making them difficult to disperse uniformly [11,12,13]. Additionally, their weak interfacial synergy with cementitious matrices often results in limited reinforcement effects [11,14]. Research indicates that for lightweight, nanoscale CNTs to fully realize their potential, they must be uniformly distributed within a lightweight, homogeneous matrix [15,16].

Polyvinyl alcohol (PVA) offers advantages such as a light weight, excellent uniformity, low cost, and good compatibility with carbon nanotubes. Previous studies demonstrated that CNTs can be uniformly dispersed in PVA colloids, significantly enhancing the physical and mechanical properties of PVA films [17]. Coleman et al. [18] synthesized CNTs/PVA nanocomposite films using a solvent evaporation method and found that with a CNT content of 0.06%, the tensile strength, Young’s modulus, and fracture toughness of the PVA film increased by 330%, 270%, and 70%, respectively. Similarly, Ryan et al. [19] prepared CNTs/PVA films via a drop-casting method and observed that at a 0.06% CNT loading, the tensile strength and Young’s modulus of the PVA film improved by 61.5% and 480%, respectively. Additionally, through gelation processing, PVA can form a three-dimensional semi-crosslinked film structure [17], which enhances the mechanical and durability properties of cementitious materials. Currently, PVA is widely used in concrete as a modifier, aggregate surface pretreatment agent, and reinforcing fiber [20,21]. Compared with conventional cement mortar, the optimized PVA-CNTs composite exhibited substantially improved mechanical performance [22], which showed a 53% higher compressive strength and doubled (101% increase) both the tensile strength and fracture toughness at 28 days. Moreover, CNTs/PVA can improve the steel protection performance of concrete, mitigating the electrochemical corrosion of reinforcement [23].

Previous studies have demonstrated that appropriate doses of CNTs can enhance certain properties of cement-based materials [24,25,26]. Reis et al. [27] demonstrated that isopropanol-pre-dispersed CNTs (0–0.10 wt%) significantly enhanced the concrete performance, where a 0.05% addition reduced the porosity by 12% and increased the tensile strength by 29%. Wang et al. [28] revealed that carbon nanotube sponge powder (CNTSPPs) under steam curing formed a high-performance CNT/hydrate nanocomposite shell, which boosted the 28-day compressive strength by 16% at 0.05 wt%. Tafesse et al. [29] found that CNTs primarily act as micro-fillers in cement matrices, altering the shrinkage behavior without affecting the hydration, though low doses may increase the autogenous shrinkage. Kim et al. [30] have reported CNTs’ ability to inhibit early-age cement hydration, thereby reducing shrinkage. Song et al. [31] discovered that silica fume significantly improved CNT dispersion in mortar by decreasing the shrinkage by 22%. Wang et al. [32] developed a novel hydrophilic CNT sponge (H-CNTSP) internal curing agent that simultaneously suppressed shrinkage and enhanced the compressive strength at 0.05–0.2 wt% through nanocomposite shell formation. Li et al. [33] confirmed that 0.05 wt% CNTs reduced paste shrinkage by 22.1%. While PVA is commonly used as a dispersant for CNTs, limited research exists on their combined effects on concrete performance. Wang et al. [23] have reported that hybrid CNTs-OH/PVA (0.5%/1%) enhanced rebar corrosion resistance by 94% and strength by 20%. Fan et al. [34] investigated the mechanical properties of CNT-PVA modified cement mortar. Ömer Güler [35] found that water-soluble-surfactant-modified (e.g., with PVA) CNTs significantly enhanced the concrete strength (>60% improvement at 0.05 wt%). Previous studies primarily focused on cement paste or mortar, with limited research on concrete applications. Moreover, while the mechanical properties and autogenous shrinkage have been extensively investigated, drying shrinkage has received considerably less attention in the literature. Notably, previous research predominantly employed expensive, high-purity CNTs. This study focused on investigating the synergistic effects of cost-effective, industrial-grade, hydroxyl-functionalized CNTs with PVA on the drying shrinkage behavior of concrete.

## 2. Materials and Methods

### 2.1. Materials

The cement used was Portland cement (P.O. 42.5 R) produced by the Guangdong Tapai Group. The fine aggregate consisted of natural river sand (fineness modulus = 2.5), with crushed stone (D_max_ = 10 mm) as the coarse aggregate. The quality and testing methods for sand and stone complied with the JGJ52-2006 standard [36].

PVA (Xilong Chemical, Shantou, China) and hydroxylated MWCNTs (Chengdu Organic Chemicals, CAS, Chengdu, China) were used, with properties detailed in Table 1 and Table 2, respectively.

### 2.2. Sample Preparation

This study designed five experimental groups with mix proportions as shown in Table 3, including ordinary concrete (C0), PVA-modified concrete (P0), and CNTs/PVA composite-modified concrete (with CNT doses of 0.3%, 0.5%, and 1.0%, respectively, and a fixed PVA dose of 1.0%). The experimental program consisted of three parts: (1) For the mechanical properties evaluation, 15 prismatic specimens (100 mm × 100 mm × 400 mm) were prepared, with 3 replicates for each of the 5 mix designs; (2) for the drying shrinkage assessment, another 15 prismatic specimens (100 mm × 100 mm × 515 mm) were cast following the same replication scheme; (3) for the capillary water absorption testing, 15 cubic specimens (70.7 mm × 70.7 mm × 70.7 mm) were prepared, also with 3 replicates per mix design. Here, CNTs, PVA, and the water-reducing agent were all calculated as percentages of the cement mass. During specimen preparation, the following equipment was used: constant-temperature water bath (Model HH-8S, Miqi Instruments Co., Ltd., Changsha, China): power 1500 W and frequency 50 Hz; ultrasonic cleaner (Model AK-040D, Shenzhen Xinkaida Electronics Co., Ltd., Shenzhen, China): power 480 W and frequency 40 kHz.

**Figure 1:**
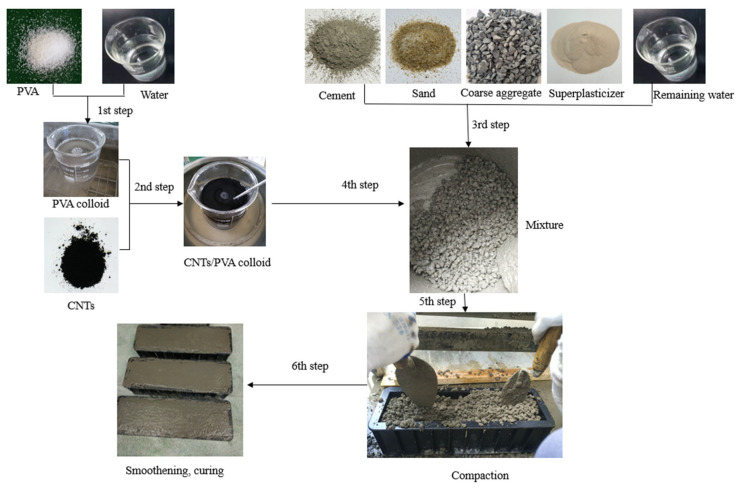
Fabrication process of CNTs/PVA modified concrete specimens.

The concrete specimen preparation steps were as follows (Figure 1):

(1) Dissolve PVA in water at 95 °C and stir it into a uniform colloid using a magnetic stirrer (approximately 30 min).
(2) Add CNTs to the PVA sol and ultrasonically disperse the carbon nanotubes at 60 °C using an ultrasonic device (1.5 h) to form a uniformly dispersed CNTs/PVA solution.
(3) Place cement, sand, and aggregates into a mixer and dry mix for 1 min.
(4) Add the CNTs/PVA solution, remaining water, water-reducing agent, and defoamer into the mixer and stir for 5 min.
(5) After mixing, pour the concrete into oil-coated molds and compact it on a vibrating table.
(6) Level the surface of the concrete in the molds, cover them with wet cloth for curing, demold after 24 h, and then place the specimens in water for further curing.

### 2.3. Performance Testing

The concrete mechanical performance was evaluated following the GB/T 50081-2019 test standards for ordinary concrete [37].

Flexural strength test: A CBT1305 microcomputer-controlled universal testing machine produced by MTS Industrial Systems (China) Co., Ltd. (Shanghai, China) was used, with a loading rate of 0.05 mm/min. Each mix proportion included three specimens, and the flexural strength was taken as the average value. Compressive strength test: A YAW4306 microcomputer-controlled pressure testing machine produced by MTS Industrial Systems (China) Co., Ltd. was employed, with a loading rate of 0.5 MPa/s. Each mix proportion included six specimens, and the compressive strength was taken as the average value.

Concrete drying shrinkage was evaluated in accordance with the Chinese national standard GB/T 50082-2009 for long-term durability testing [38]. Each mix proportion included three specimens that measured 100 mm × 100 mm × 515 mm, and the testing instrument was an HSP-540 concrete shrinkage–expansion apparatus that was manufactured by Zhiyou Instrument Equipment Co., Ltd. (Shaoxing, China).

Capillary absorption rate test method: The specimens for the capillary absorption rate testing were cubic specimens with dimensions of 100 mm × 100 mm × 100 mm. The unit water absorption of the mortar specimens was calculated using Formula (1), and the capillary absorption rate was obtained by calculating the slope from the linear correlation:

(1) $$\frac{Q}{A}=k\cdot \sqrt{t}$$

where *Q* is the cumulative water absorption of the specimen in grams (g), *A* is the surface area of the specimen available for water absorption in square meters (m^2^), *t* is the cumulative water absorption time of the specimen in minutes (min), and *k* is the capillary absorption coefficient of the specimen g/(m^2^·min^1/2^).

Following the compressive strength testing, representative concrete specimens were randomly selected for subsequent microstructural characterization using scanning electron microscopy (SEM). The SEM tests were performed using a Zeiss Gemini 300 (Carl Zeiss AG, Oberkochen, Germany).

## 3. Results

### 3.1. Mechanical Properties

**Figure 2:**
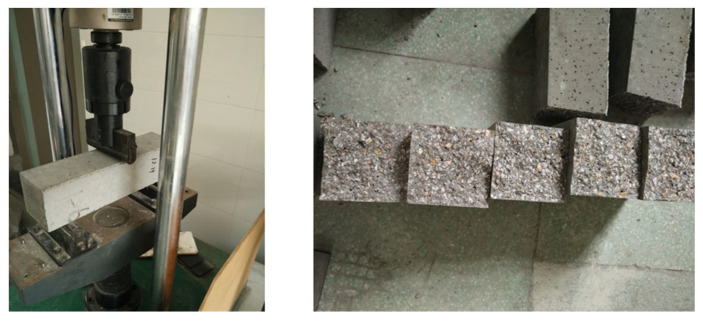
Post-failure specimens of the concrete samples.

Figure 2 presents the mechanical performance test results and post-failure specimens of the concrete samples. As shown in this figure, the failure surfaces of the plain concrete specimens were generally flat and smooth. In contrast, the specimens that incorporated CNTs and PVA exhibited more irregular fracture surfaces. Notably, the addition of CNTs reduced the presence of large pores on the fracture surface, suggesting improved microstructure integrity. This observation indicates that the CNT and PVA modifications altered the failure mechanism and enhanced the material’s internal structure. Following compressive strength testing, representative concrete specimens were randomly selected for subsequent microstructural characterization using scanning electron microscopy (SEM).

**Figure 3:**
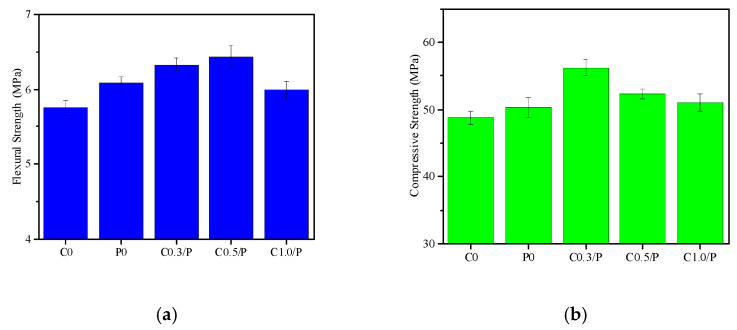
Mechanical performance of the concrete: (**a**) flexural strength; (**b**) compressive strength.

Figure 3 presents the flexural and compressive strength test results of the concrete with different mix proportions after 28 days of curing. The results indicate that the incorporation of PVA alone could improve the mechanical properties of concrete, but the enhancement effect was relatively weak. Compared with ordinary concrete, the addition of 1.0% PVA only increased the 28-day compressive and flexural strengths by 3% and 6%, respectively.

In contrast to the single incorporation of PVA, the combined use of CNTs/PVA demonstrated significantly more pronounced enhancement effects. As shown in Figure 3a, regarding flexural strength, the C0.5/P concrete group exhibited superior mechanical properties, where it achieved a 28-day flexural strength of 6.5 MPa—12% higher than that of the ordinary concrete. When the CNTs content was 0.3% and 1.0%, the 28-day flexural strength increased by 10% and 4%, respectively. Figure 3b reveals that for the compressive strength, the C0.3/P group displayed an optimal mechanical performance, where its 28-day compressive strength increased from 48.8 MPa to 56.2 MPa—a 15% improvement. The compressive strengths of the C0.5/P and C1.0/P concrete groups increased by 7% and 5%, respectively. The flexural strength ANOVA (F*(4,10) = 5.82, *p* = 0.011) identified the C0.5/P group as the top performer (12.0% higher than the controls). For the compressive strength, the C0.3/P formulation achieved a 15.2% boost (*F*(4,25) = 32.17, *p* < 0.001), which demonstrated CNTs/PVA’s dose-dependent effects.

As can also be observed from Figure 3, when the CNTs content reached 1.0%, both the compressive and flexural strengths of concrete showed unsatisfactory performance. This phenomenon can be attributed to the fact that with an excessive CNT dose, the relative proportion of PVA became insufficient in the system. Under such conditions, the colloidal stability and interfacial bonding forces provided by PVA were inadequate to overcome the van der Waals forces between the CNTs, resulting in CNT aggregation that severely compromised their modification effectiveness. Furthermore, comparative analysis with reference [22] revealed that the enhancement effect of CNTs and PVA on the concrete’s mechanical properties was significantly inferior to their effect on cement mortar. This finding suggests that coarse aggregates substantially diminish the reinforcing capability of CNTs and PVA in concrete systems.

### 3.2. Drying Shrinkage

**Figure 4:**
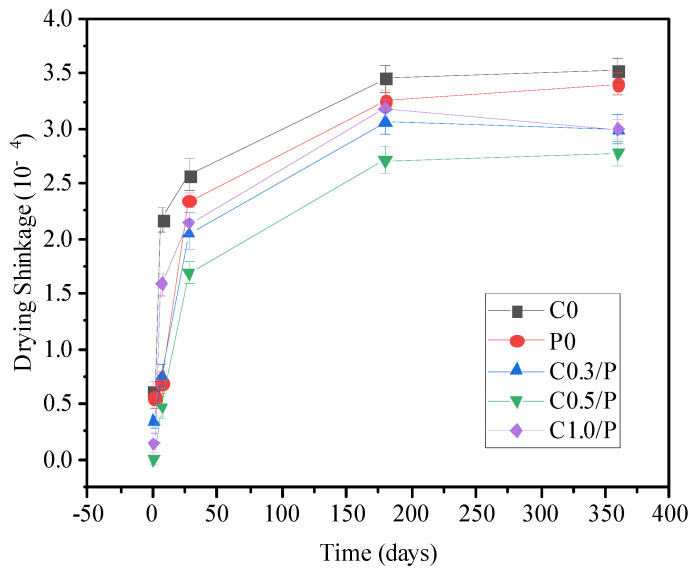
Influences of CNTs/PVA on drying shrinkage of concrete.

Figure 4 shows the drying shrinkage test results of the concrete cured for 1 to 360 days. By comparing the drying shrinkage curves of the C0 and P0 groups, it can be observed that as the curing age increased, the drying shrinkage rates of both the ordinary concrete and PVA-modified concrete increased. The shrinkage rate changed rapidly in the early stage (before 28 days) but slowed down and stabilized in the later stage (after 180 days). Figure 3 also indicates that the incorporation of PVA alone could reduce the drying shrinkage of concrete at all ages, with the effect being more pronounced at shorter curing ages. The drying shrinkage rate of the C0 group increased by 323% after 28 days of curing compared with 1 day, while the increase was only 2% after 360 days compared with 180 days. The drying shrinkage rate of the P0 group was generally lower than that of the ordinary concrete at all tested ages. However, the growth rate of the drying shrinkage in the P0 group with increasing age was significantly higher than that of the ordinary concrete. Relative to the 1-day curing age, the drying shrinkage rates of the P0 group increased by 25%, 132%, 364%, 454%, and 507% after 7, 60, 180, 270, and 360 days of curing, respectively.

Furthermore, Figure 4 also demonstrates that the combined incorporation of CNTs and PVA could further reduce the drying shrinkage of concrete. The variation trends in the shrinkage rates were very similar—both increased with curing age, exhibited rapid changes in the early stage and slowed down in the later stage, and stabilized after 180 days. Additionally, Figure 4 indicates that the dose of CNTs also influenced the drying shrinkage of concrete. Among all the tested ages, the C0.5/P group exhibited the lowest drying shrinkage rate. Moreover, a comparison of the drying shrinkage results between the C0.5/P and C0 groups revealed that after 7, 28, 150, 180, 270, and 360 days of curing, the drying shrinkage rates of the C0.5/P group were reduced by 76%, 34%, 29%, 22%, 22%, and 21%, respectively, compared with the C0 group. These test results confirm that the combined use of CNTs and PVA could effectively mitigate the drying shrinkage of concrete. Moreover, after 180 days of curing, the drying shrinkage performance of the concrete essentially stabilized. The drying shrinkage ANOVA confirmed statistically significant differences between the groups (F(4,45) = 18.32, *p* < 0.001). The C0.5/P group showed a 21–22% reduction in the long-term shrinkage (180–360 days) compared with the control group (C0) (*p* < 0.01), which significantly outperformed the PVA-only modified concrete (P0 group, 3–6% reduction).

### 3.3. Capillary Water Absorption

**Figure 5:**
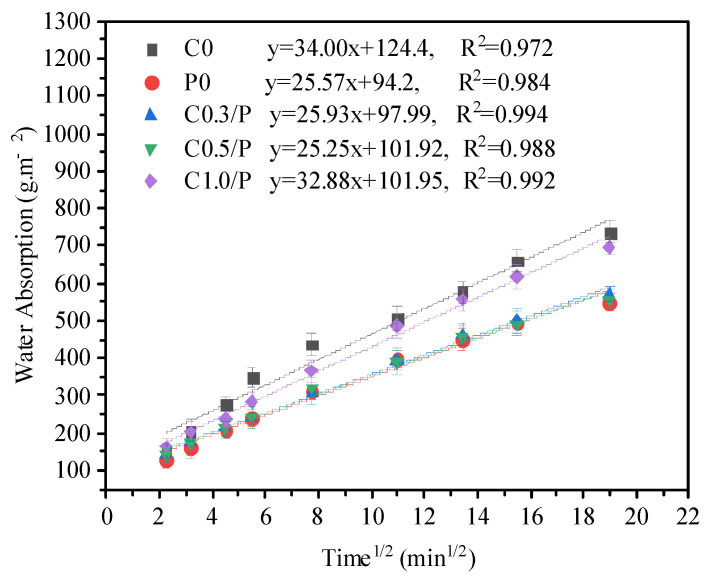
Influences of CNTs/PVA on water absorption of concrete.

Figure 5 illustrates the capillary water absorption behavior of the CNT- and PVA-modified concrete. The results show that the ordinary concrete exhibited a high capillary water absorption rate, with a capillary absorption coefficient of 34.00 g/m^2^. However, after incorporating the PVA, the coefficient decreased to 25.57 g/m^2^. This reduction was primarily attributed to the film-forming property of PVA upon dehydration—the resulting film structure filled and segmented the pores, which made them discontinuous, and thus, reduced the capillary water absorption. When the CNT content was low, they dispersed uniformly within the PVA matrix, which led to a lower capillary absorption rate. This was because the well-dispersed CNTs/PVA formed a network structure that enhanced the internal microstructure of the concrete. Additionally, the CNTs contributed to pore refinement by filling the voids. However, when the CNTs content reached 1.0%, the capillary absorption coefficient increased to 32.88 g/m^2^, which showed almost no reduction compared with the ordinary concrete. This was mainly due to excessive CNT aggregation, which hindered effective dispersion by the PVA. As a result, the improvement in the concrete performance became negligible. For capillary water absorption, the ANOVA revealed significant variations (F(4,15) = 9.87, *p* = 0.002). The C0.5/P composite achieved a 26% lower absorption rate (25.25 vs. 34.00 g/m^2^, *p* < 0.001) than the plain concrete (C0), which demonstrated its superior pore structure refinement.

### 3.4. Microstructure

**Figure 6:**
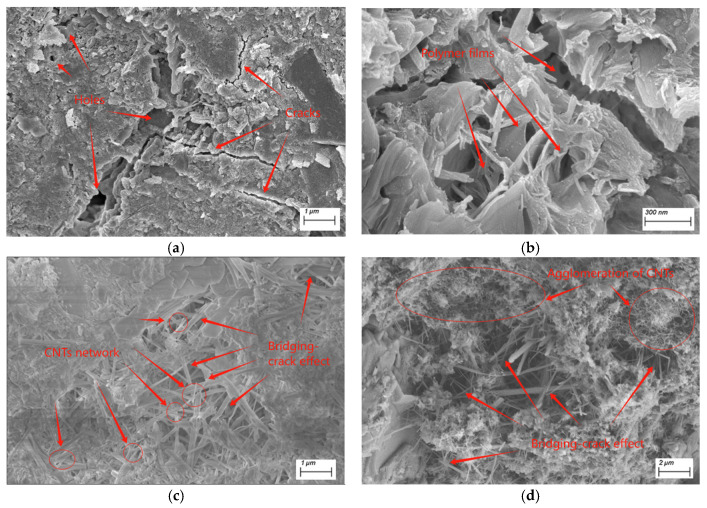
Influences of CNTs/PVA on microstructures of concrete. (**a**) C0; (**b**) P0; (**c**) C0.5/P; (**d**) C1.0/P.

Figure 6a presents the microscopic morphology of ordinary concrete, revealing the presence of brittle cracks and numerous pores. These cracks and voids not only reduced the load-bearing capacity of concrete but also accelerated the transport of various ions and water molecules, which negatively impacted its safety and durability. Figure 6b displays the microstructure of the PVA-modified concrete, where a distinct film structure can be observed. This film bridged the ends of the cracks, which effectively inhibited crack propagation.

Figure 6c shows the microstructure of the C0.5/P modified concrete. The image reveals a dense microstructure with flexible fibers that interpenetrated the cement paste and formed bridging connections that effectively transferred stress and inhibited crack propagation. Numerous flexible network structures spanned across microcracks in the cement matrix, which not only sealed and refined the pores but also bridged hydration products, which created a fine “hoop effect” that enhanced the structural integrity. Additionally, the flexible fibers were uniformly dispersed without visible agglomeration, indicating that a low CNT content (0.5%) combined with colloidal stability and interfacial chemical interactions ensured effective dispersion. The observed fiber diameters were significantly larger than those of the pristine CNTs, which confirmed that each CNT was coated with a PVA film, demonstrating excellent compatibility and synergistic reinforcement between the PVA and CNTs.

Figure 6d presents the microstructure of the C1.0/P group. While the PVA-wrapped CNT fibers still exhibited bridging effects, CNT agglomerations were clearly visible due to the excessive dose (1.0%), which led to an uneven dispersion and particle clustering. This compromised the intended reinforcing mechanism.

## 4. Discussion

Several studies [39] have reported that prolonged ultrasonication may cause length reduction and defect generation in CNTs. However, in the present study, this issue was effectively avoided, as evidenced by the following three aspects: (1) Protective role of the PVA matrix: In the method, CNTs were first dispersed in a viscous polyvinyl alcohol pre-polymer solution before the ultrasonication. The high viscosity of the PVA significantly attenuated the ultrasonic energy transmission to the CNTs. This shielding effect minimized direct cavitation-induced damage, unlike in aqueous or solvent-based systems, where CNTs are exposed to unfiltered ultrasonic energy. (2) Microstructural evidence (Figure 6c,d): The SEM images (Figure 6c) confirmed that the CNTs remained embedded within the PVA matrix and formed a continuous network without visible fragmentation. The presence of CNT agglomerates (Figure 6d) further suggests that the ultrasonic energy was insufficient to fully disentangle all the bundles, indirectly supporting limited structural damage. (3) Macroscopic performance validation: the enhanced mechanical properties (e.g., 15% increase in compressive strength) and reduced drying shrinkage of the composite empirically validated the effective CNT dispersion and interfacial bonding, which would be unlikely if severe CNT fragmentation or defect generation had occurred.

Based on the experimental results mentioned above, it can be concluded that the single incorporation of PVA can improve the mechanical properties of concrete. This is because PVA can form a polymer film within the concrete [22], which helps seal pores and restrain the formation, propagation, and interconnection of brittle cracks in the concrete. Additionally, the -OH groups in PVA can react with cement hydration products to form chemical bonds, thereby further enhancing the mechanical properties of the concrete. However, due to the extremely low dose of PVA, the 28-day compressive and flexural strengths of PVA-modified concrete showed only marginal improvement.

When an appropriate amount of CNTs and PVA are co-incorporated into concrete, they become uniformly dispersed and form a three-dimensional network structure (as shown in Figure 6c), effectively bridging cracks (Figure 6c) and refining pores. Combined with the surface effect and morphological effect of CNTs [40,41,42,43], both C0.3/P and C0.5/P enhanced the compressive and flexural strength of concrete, as illustrated in Figure 2. Existing studies have also demonstrated that CNTs can significantly enhance the mechanical properties of cement-based materials. Reis et al. [27] reported increases of 29% in flexural strength and 16% in compressive strength. Ashwini [44] observed strength improvements of 27% (compressive) and 7% (flexural), while Kim et al. [45] documented a maximum compressive strength enhancement of up to 66%. Under elevated temperatures, Gao et al. [46] verified that the compressive strength improvement ranged from 5% to 65%. Fan et al. [34] reported the compressive/flexural strengths of the modified mortar increased by 16%/42%. However, when the CNTs content is excessive, the relatively low proportion of PVA in the system fails to utilize colloidal stability and interfacial forces to overcome the van der Waals forces between CNTs. As a result, a large number of CNTs remain in an aggregated state (Figure 6d), leading to suboptimal reinforcement. Additionally, it is worth noting that the strengthening effect of CNTs and PVA on the mechanical properties of concrete is significantly lower than that in cement mortar. This discrepancy may be attributed to the relatively low doses of CNTs and PVA used in this study, which may not yet form an effective stress-transfer network in concrete. The exact reasons require further investigation.

The drying shrinkage deformation of concrete is primarily related to the migration rate and volume of physically adsorbed water in the C-S-H gel and capillary-adsorbed water in the cement paste. Generally, the faster the migration rate and the greater the volume of adsorbed water, the higher the drying shrinkage rate and magnitude of concrete [47]. Since PVA can form sheet-like films in concrete (Figure 6b), which seal pores and cracks, it effectively delays the migration of mixing water inside the concrete. Therefore, the incorporation of PVA alone can reduce the early-age (before 28 days) drying shrinkage rate of concrete. However, as the curing age increases, the mixing water in PVA-modified concrete gradually decreases due to cement hydration and changes in environmental humidity. Additionally, PVA films themselves exhibit significant shrinkage upon drying and expansion upon wetting. As a result, the drying shrinkage rate of PVA-modified concrete eventually surpasses that of ordinary concrete. As shown in Figure 3, the drying shrinkage of PVA-modified concrete approached that of ordinary concrete at 360 days.

In contrast, the combined use of CNTs and PVA can effectively reduce the drying shrinkage of concrete: (1) The -OH groups in the CNTs-PVA system can chemically adsorb cement hydration products, reducing the content of physically adsorbed water in the C-S-H gel and restraining strain generation in the C-S-H gel under humidity gradients, thereby lowering the concrete drying shrinkage. (2) CNTs and PVA act as pore fillers, refining and reducing the porosity of concrete, which decreases the internal pore water content and avoids drying shrinkage deformation caused by capillary water migration. (3) The fibrous CNTs and PVA form a network structure in concrete (Figure 6c), exhibiting a long-fiber effect and a micro-confinement effect. This structure prevents the formation, propagation, and interconnection of cracks (which serve as pathways for moisture escape), thereby reducing the drying shrinkage and enhancing the safety and durability of concrete structures.

## 5. Conclusions

(1) Scanning electron microscopy (SEM) tests indicated that an appropriate amount of CNTs and PVA was uniformly dispersed in the concrete and formed a three-dimensional linear interpenetrating network. This network refined pores, bridged cracks, and improved the micro- and meso-scale properties of the concrete.
(2) The CNT/PVA synergy significantly enhanced the mechanical properties: with 1.0% PVA, (1) 0.5% CNTs elevated the flexural strength by 12% (*p* < 0.01 via Tukey’s test), and (2) 0.3% CNTs boosted the compressive strength by 15% (*p* < 0.001) versus plain concrete controls, demonstrating a dose-dependent reinforcement.
(3) The drying shrinkage rate of CNTs and PVA composite-modified concrete increased with age, but it remained essentially unchanged after the curing age exceeded 180 days. The CNTs and PVA could reduce the drying shrinkage rate of concrete. When the CNTs content was 0.5%, the drying shrinkage rates at 7, 28, 180, and 360 days decreased by 76%, 34%, 22%, and 21%, respectively.
(4) The CNT/PVA modification (1.0% PVA + 0.5% CNTs) achieved a 26% reduction in the capillary absorption coefficient versus the control concrete (*p* < 0.01), which demonstrated enhanced water resistance.
(5) The addition of PVA alone could reduce the early drying shrinkage of concrete, but the shrinkage rate increased rapidly. At 360 days of age, the drying shrinkage rate of the PVA-modified concrete was nearly the same as that of ordinary concrete.
(6) When PVA was used alone or when the CNTs content was excessive (e.g., 1.0%), the improvement in the 28-day strength of concrete was not significant. With PVA alone or a composite mix of 1.0% CNTs, the strength of the concrete increased by only 3–6%.

## Figures and Tables

**Table 1:** Physicochemical characteristics of PVA.

Molecular Weight/(*M_w_*)	Degree of Hydrolysis/(mole%)	pH	Volatile Content/(%)	Ash Content/(%)
105,000	97	5–7	5.0	0.7

**Table 2:** Properties of hydroxyl CNTs.

Diameter/(nm)	Length/(µm)	Purity/(wt%)	Special Surface Area/(m^2^/g)	True Density/(g/cm^3^)	-OH Content/(wt%)
10–30	30–50	>90	>230	2.1	5.58

**Table 3:** Mix proportions of modified concrete.

	Cement/(kg/m^3^)	Water/(kg/m^3^)	Sand/(kg/m^3^)	Gravel/(kg/m^3^)	PVA/(%)	CNTs/(%)	Superplasticizer/(%)
C0	380	171	593	1186	0	0	0.2
P0	380	171	593	1186	1	0	0.4
C0.3/P	380	171	593	1186	1	0.3	0.6
C0.5/P	380	171	593	1186	1	0.5	0.8
C1.0/P	380	171	593	1186	1	1.0	1.0

## Data Availability

The original contributions presented in this study are included in the article. Further inquiries can be directed to the corresponding author.
